# Nonlinear Aging of the Human TCR Repertoire Is Influenced by Sex, CMV, and Cancer

**DOI:** 10.1111/acel.70777

**Published:** 2026-10-25

**Authors:** Naja Lange, Asbjørn K. Attermann, Randi Istrup Juul, Laura Andersen, Iver Nordentoft, Leon Eyrich Jessen, Nicholas McGranahan, Hugo J. W. L. Aerts, Lars Dyrskjøt, Nicolai J. Birkbak

**Affiliations:** ^1^ Department of Molecular Medicine (MOMA) Aarhus University Hospital Aarhus Denmark; ^2^ Department of Clinical Medicine Aarhus University Aarhus Denmark; ^3^ Department of Health Technology, Section for Bioinformatics Technical University of Denmark, DTU Lyngby Denmark; ^4^ Cancer Research UK Lung Cancer Centre of Excellence University College London Cancer Institute London UK; ^5^ Cancer Genome Evolution Research Group University College London Cancer Institute London UK; ^6^ Artificial Intelligence in Medicine (AIM) Program Mass General Brigham, Harvard Medical School Boston Massachusetts USA; ^7^ Department of Radiation Oncology Brigham and Women's Hospital and Dana‐Farber Cancer Institute, Harvard Medical School Boston Massachusetts USA; ^8^ Radiology and Nuclear Medicine GROW & CARIM, Maastricht University Maastricht the Netherlands

**Keywords:** aging, biomarkers, cancer, cytomegalovirus, immune aging, immunosenescence, T cell receptor, TCR sequencing

## Abstract

Aging is accompanied by a progressive decline in immune function, increasing susceptibility to infections, cancer, and poorer treatment outcomes. The diversity of the T cell receptor (TCR) repertoire, reflecting the capacity to recognize diverse antigens, is known to decline with age. However, how intrinsic and extrinsic factors jointly shape TCR diversity and its rate of decline, as well as the extent to which this diversity influences clinical outcomes in cancer, remain poorly characterized. Here, we integrate TCR sequencing data from 1.32 billion unique T cell clones, derived from 798 patients with cancer and 5052 individuals without cancer across 14 independent cohorts, to systematically characterize the effects of age, sex, and chronic cytomegalovirus (CMV) infection on peripheral blood repertoire diversity. We identify a nonlinear trajectory of diversity loss that accelerates after midlife, with a marked increase in the rate of decline beyond approximately 50 years of age, which is further modified by sex and CMV infection. In patients with cancer, higher peripheral TCR diversity was associated with improved overall survival across multiple cancer types, even when accounting for the impact of age, sex, and CMV on diversity. Together, these findings underline peripheral TCR repertoire diversity as a dynamic marker of immune aging shaped by demographic and chronic viral factors.

## Graphical abstract

Aging is associated with a nonlinear decline in peripheral blood TCR repertoire diversity, with an accelerated decline after ~50 years, while sex, CMV infection, and cancer further shape TCR diversity. The age‐associated decrease in diversity was accompanied by shifts in clonal and T cell subtype composition. Higher diversity was associated with prolonged survival in patients with cancer.

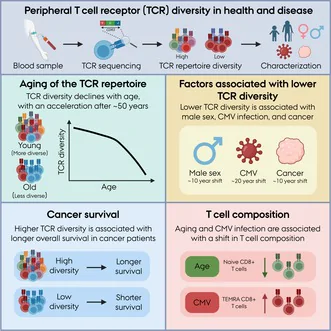

## 1 Introduction

Aging profoundly reshapes the immune system, altering its composition, function, and capacity to respond to new challenges. This process, known as immunosenescence, affects both innate and adaptive immunity and contributes to increased susceptibility to infections and impaired immune surveillance in older individuals (Deeks 2011). One of the central drivers of immunosenescence is thymic involution, the age‐associated shrinkage and functional deterioration of the thymus, which progressively limits the production of naïve T cells (Mittelbrunn and Kroemer 2021). Thymic function varies substantially across adulthood, and preserved activity has been linked to improved health outcomes, underscoring the importance of sustained T cell generation for immune competence (Bernatz, et al. 2026a, 2026b). As thymic output declines, the T cell compartment becomes increasingly dominated by memory T cells, and immune maintenance becomes more dependent on peripheral expansion of existing T cell clones (Liang et al. 2022). These changes limit the immune system's ability to recognize new antigens, including those derived from previously unencountered pathogens as well as tumor‐specific neoantigens (Aw et al. 2007). Consistent with this, cancer is a strongly age‐associated disease and has been hypothesized to arise partly due to immunosenescence later in life (Dolan et al. 2025).

T cells are key components of the adaptive immune system and play a central role in recognizing and eliminating pathogens and cancerous cells. Antigen recognition is mediated by the surface‐bound T cell receptor (TCR) (Farhood et al. 2019). The TCR is a heterodimeric protein consisting of an ɑ‐ and a β‐chain, which is generated through V(D)J somatic recombination, whereby a unique receptor for each T cell clone is produced (Greenaway et al. 2013). This process underlies the vast diversity of TCR repertoires that are essential for effective T cell‐mediated immunity (Glanville et al. 2017). Upon encountering its cognate antigen, a naïve T cell undergoes rapid clonal expansion, generating numerous daughter cells with identical TCR sequences to mount an effective immune response (Thapa and Farber 2019). An effective T cell response, therefore, depends on a highly diverse TCR repertoire capable of recognizing a wide range of antigens. However, this repertoire is dynamic and changes throughout life, influenced by infections (Grabauskas et al. 2026), environmental exposures (Brodin et al. 2015), and sex (Márquez et al. 2020; Zahid et al. 2024) with a gradual decline in diversity observed with aging (Britanova et al. 2014). Although this age‐related decline in immune competence is well recognized, the dynamic remodeling of the TCR landscape and its potential clinical implications remain poorly characterized, particularly in the context of cancer. Thus, there is a growing need for robust metrics of immune health that can inform prognostic assessment and therapeutic decision‐making in both health and disease.

Beyond thymic involution, chronic viral infections can also significantly alter the T cell landscape, imposing substantial and lasting changes (van der Heiden et al. 2016). Latent viral infections such as cytomegalovirus (CMV) can trigger a phenomenon known as “*memory inflation*”, in which virus‐specific T cell clones undergo extensive expansion and persist at high frequencies even in the absence of active infection (Kuhn et al. 2022). In some individuals, CMV‐specific T cells can comprise up to 30% of the total memory T cell pool in peripheral blood (Zangger and Oxenius 2022). This expansion skews the T cell repertoire by reducing diversity, potentially impairing the ability to mount an effective immune response against new pathogens or tumor antigens (Derhovanessian et al. 2014).

Profiling the composition and diversity of the TCR repertoire provides a window into the functional state of the immune system. High‐throughput TCR sequencing (TCR‐seq) assays enable such characterization, commonly by targeting the complementarity‐determining region 3 (CDR3) of the TCR β‐chain, the most variable segment of the TCR that spans the V(D)J junction and determines antigen specificity (Chronister et al. 2021). In this way, TCR‐seq captures key features of immune competence, including the balance between naïve and memory compartments, the extent of antigen‐driven clonal expansions, and potential deficits in repertoire breadth. These features may hold significant value for understanding immunosenescence and for predicting clinical outcomes in cancer.

While age, sex, and CMV infection are known to influence the TCR landscape, their combined impact on repertoire structure in health and cancer remains incompletely defined. In this study, we assembled TCR‐seq data from 14 cohorts comprising both individuals without cancer and patients with cancer to systematically characterize the impact of age, sex, and CMV status on TCR repertoire diversity and clonal composition (Figure 1a). We further evaluated the prognostic relevance of TCR diversity across multiple cancer types, including an independent analysis of The Cancer Genome Atlas (TCGA) cohort using TCR estimates derived from whole‐exome sequencing (WES) data. These integrated analyses provide new insights into how age and chronic viral infection shape the immune repertoire dynamics and how these factors influence cancer outcomes.

**FIGURE 1:**
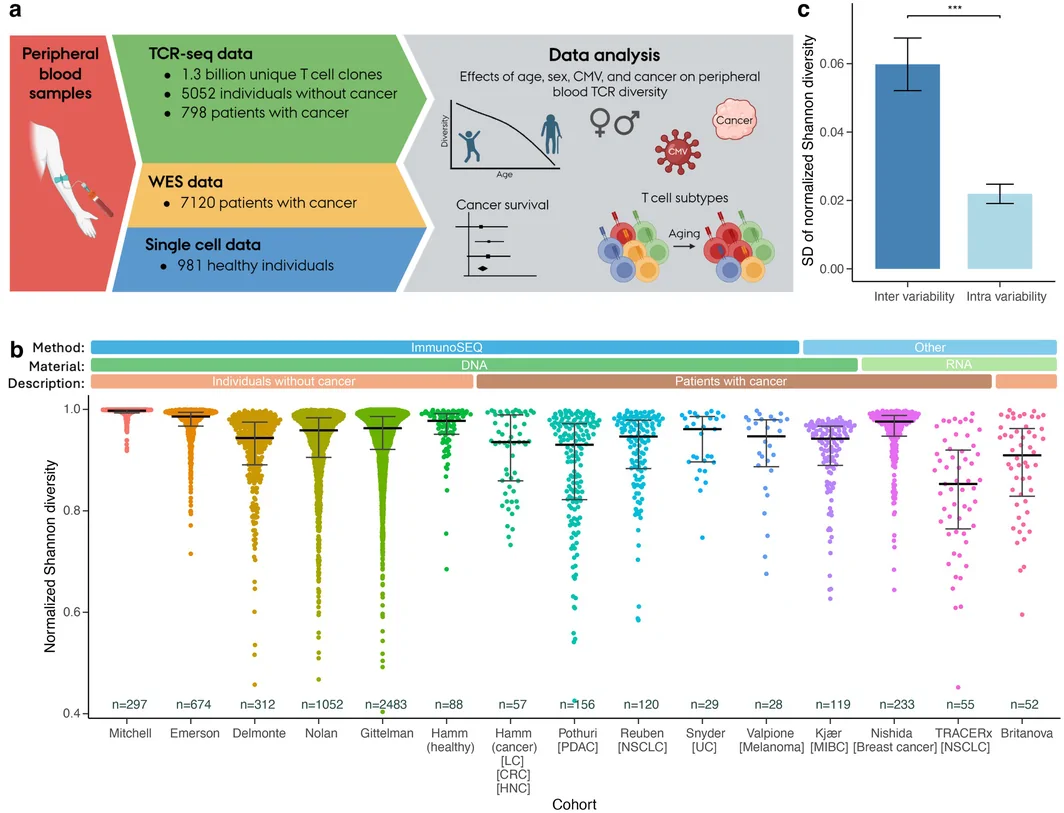
Study design and data overview. (a) Overview of the study analyses. The figure was created with BioRender. Birkbak, N. (2026) BioRender.com/6err5uw. (b) Distribution of normalized Shannon diversity values stratified by cohort. Cohorts are grouped by sequencing method, input material, and cohort type. The five largest cohorts comprising individuals without cancer are ordered by increasing age span (from Mitchell, with the narrowest age range, to Gittelman, with the widest). Sample sizes (*n*) are shown below each cohort. For each dataset, the thick black horizontal line represents the median, and error bars denote the interquartile range. Cancer type is indicated in square brackets. CRC = colorectal cancer, HNC = head and neck cancer, LC = lung cancer, MIBC = muscle‐invasive bladder cancer, NSCLC = non‐small cell lung cancer, PDAC = pancreatic ductal adenocarcinoma, UC = urothelial carcinoma. (c) Standard deviations (SD) of normalized Shannon diversity for inter‐individual comparisons (age‐matched individuals) and intra‐individual comparisons (longitudinal samples from the same individuals). Error bars indicate 95% confidence intervals of the standard deviation. Differences in variability were assessed using a two‐sample *F*‐test (***p < 0.001).

## 2 Results

### 2.1 Patient and Sample Characteristics

To characterize the T cell landscape in health and disease, we compiled TCR‐seq data targeting the CDR3 region of the TCR‐β chain from 14 studies, representing 1.32 billion unique T cell clones from 798 patients with cancer and 5052 individuals without cancer (comprising healthy individuals and individuals with COVID‐19 or pre‐/at‐onset Type 1 diabetes, full details in Table S1). This dataset enabled a comprehensive analysis of TCR repertoire diversity and composition across individuals without cancer and patients with different cancer types.

Ten cohorts (Delmonte et al. 2024; Emerson et al. 2017; Gittelman et al. 2022; Hamm, n.d.; Mitchell et al. 2022; Nolan et al. 2020; Pothuri et al. 2024; Reuben et al. 2020; Snyder et al. 2017; Valpione et al. 2020) were sequenced using the same TCR sequencing assay (ImmunoSEQ by Adaptive Biotechnologies). The use of a shared sequencing workflow for the majority of datasets reduces platform‐related variability and improves cross‐cohort comparability. The remaining four cohorts were generated using other methodologies and were generally excluded from joint modeling throughout the study to reduce assay‐specific bias (see Section 4 for details).

### 2.2 High Variance in TCR Repertoire Diversity Between Samples

To assess TCR repertoire diversity, we calculated the normalized Shannon diversity index, which measures the richness and evenness of the repertoire (Chiffelle et al. 2020). As sequencing depth was observed to affect diversity estimates (Figure S1), all repertoires were downsampled to 5000 TCR‐β sequences per individual to ensure comparability within and across cohorts. Following this, the overall distribution of TCR diversity was largely consistent across cohorts, with median values ranging from 0.85 to 0.99 (Figure 1b). Despite this cross‐cohort consistency, substantial inter‐individual variability was observed. To investigate whether this variability reflected an intrinsically dynamic TCR repertoire, we compared inter‐ and intra‐individual variation in TCR diversity in a subset of patients with paired samples collected 7 months apart (*n* = 117). Inter‐individual variability was substantially greater than intra‐individual variability (*p* < 0.001, Figure 1c). This indicates that TCR diversity is relatively stable on a short timescale and that the observed inter‐individual variability is unlikely to arise from temporal fluctuations alone. Additionally, the Mitchell cohort, comprising young individuals within a narrow age range, exhibited particularly low variance in diversity. Together, these findings suggest that the observed differences in diversity likely reflect a heterogeneous TCR landscape shaped by lifelong pathogen exposures, health status, immune history, and age‐associated remodeling of the T cell compartment.

### 2.3 Nonlinear Age and Sex Effects Shape TCR Repertoire Diversity

To investigate how TCR repertoire diversity changes with increasing age, we pooled all cohorts comprising individuals without cancer that were generated using the same platform (ImmunoSEQ: Mitchell, Emerson, Delmonte, Gittelman, Nolan and Hamm [healthy subset]). Additionally, individuals were grouped into 10‐year age bins representing the level of age resolution available across all cohorts. We observed a significant age‐associated decline in repertoire diversity (Figure 2a), consistent across cohorts (Figure S2) and in alignment with previously reported observations (Weng 2023; Zahid et al. 2024). Notably, the downward trend was steeper in older age groups, suggesting a possible acceleration in the rate of decline. We compared the rate of reduction in TCR diversity across successive age groups, revealing a faster decline beginning around age 50 (Figure 2b). A segmented linear regression model with a fixed breakpoint at age 50 confirmed a shift in the rate of decline, estimating a 2.3‐fold faster reduction in TCR diversity after age 50 (*p* < 0.0001; Figure 2c; Figure S3a). To ensure that the acceleration was not dependent on the diversity metric used, we repeated the analysis using the Gini and the Simpson indices, which showed a similar acceleration (Figure S3b–e). Together, these analyses demonstrate how systemic loss of TCR diversity is not constant throughout life, but accelerates around midlife.

**FIGURE 2:**
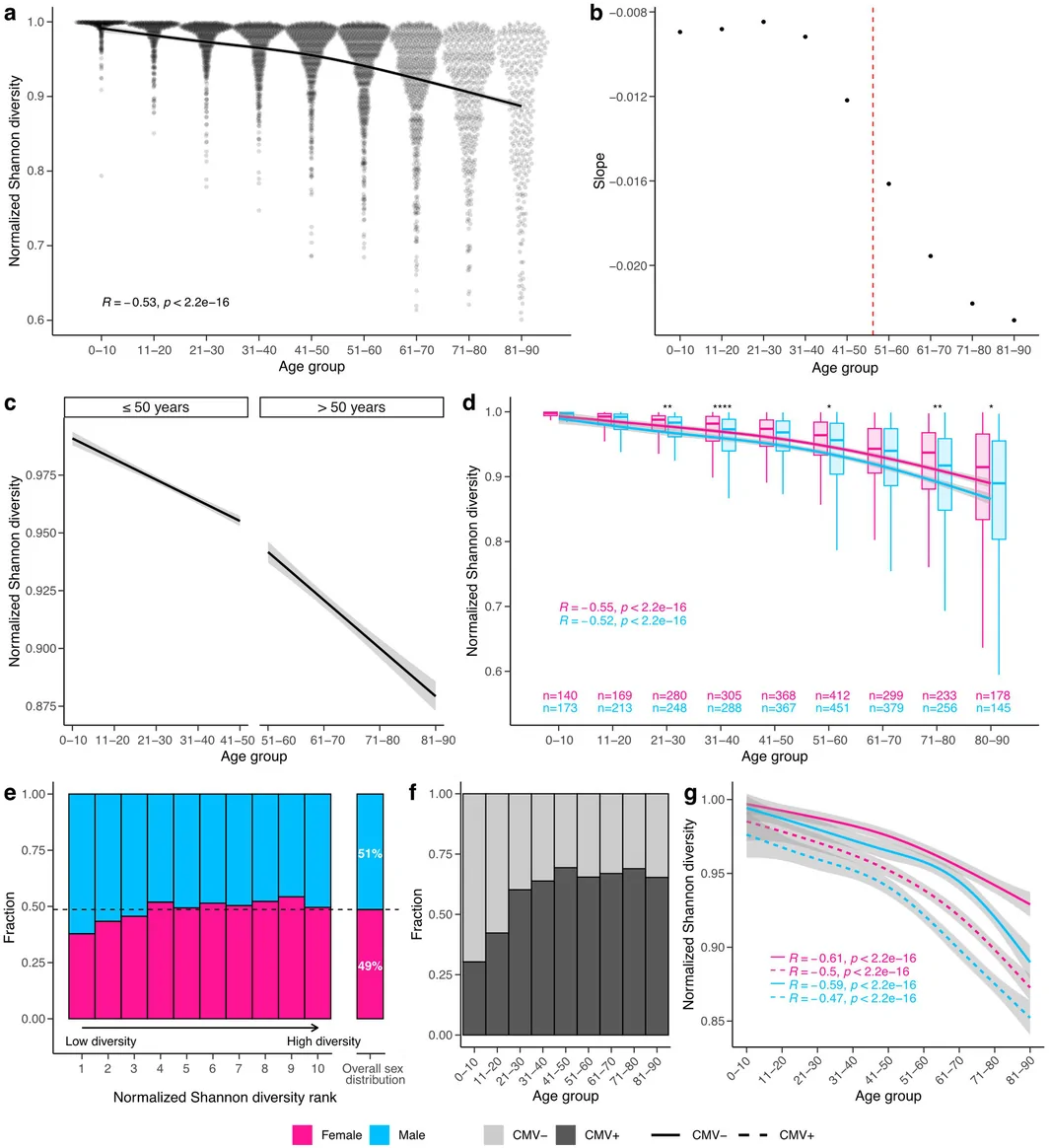
Nonlinear age‐associated decline in TCR repertoire diversity. (a) Quasirandom scatter plot showing age‐related changes in normalized Shannon diversity across 10‐year age groups. A generalized additive model (GAM) with 95% (CI) is shown in black. (b) Age‐specific slopes estimated from the GAM of normalized Shannon diversity across age groups. The vertical red dashed line marks the transition point identified by breakpoint analysis. (c) Age‐associated decline in normalized Shannon diversity shown separately for individuals ≤ 50 and > 50 years, with linear fits and 95% CIs. (d) Age‐associated decline stratified by sex. Boxplots show the distributions per age group, with GAM‐fitted curves and 95% CIs. Sample sizes (*n*) per group are shown below each box. *p*‐values were calculated using the Wilcoxon test (*p < 0.05, **p < 0.01, ****p < 0.0001). (e) Sex distribution across normalized Shannon diversity ranks. Samples were ranked by normalized Shannon diversity and divided into 10 equal‐sized bins. Bars show the fraction of males (blue) and females (pink) within each bin. The dashed horizontal line indicates the overall sex distribution in the dataset. A chi‐square goodness‐of‐fit test evaluated whether the male proportion in each bin differed from the overall cohort distribution (*χ* ^2^ = 22.1, df = 9, *p* = 0.0085). (f) CMV status distribution across age groups. The fraction of CMV+(dark gray) and CMV− (light gray) individuals is shown for each age group. (g) Age‐associated decline stratified by sex and CMV status. Curves represent GAM fits with 95% CIs. Spearman correlation coefficients and corresponding *p*‐values are shown.

We next investigated whether the observed age‐associated patterns differed between males and females (Figure 2d). Using a segmented linear regression model with a fixed breakpoint at age 50, we observed a significant effect of sex on TCR diversity in both age groups, with males showing lower diversity than females both below (*p* = 0.0004) and above age 50 (*p* < 0.0001; Figure S3f–g). Notably, this difference increased after the age of 50, similar to the effect of aging. In general, males exhibited TCR diversity comparable to that of females approximately 10 years older. Finally, stratifying individuals into 10 equal‐sized bins based on TCR diversity revealed a male‐biased enrichment among samples in the lowest diversity deciles (*p* = 0.0085; Figure 2e). Together, these findings indicate a sex‐associated skew in TCR repertoire diversity.

### 2.4 TCR Repertoire Diversity Is Influenced by CMV Status

CMV infection has previously been linked to reduced TCR repertoire diversity due to the expansion of large CMV‐specific T cell clones (Khan et al. 2002), which occupy a greater fraction of the repertoire and thereby reduce the relative abundance of other clones. However, the large influence of CMV on diversity is often overlooked in immune repertoire studies. To assess its impact, we inferred CMV status using a previously published approach (Emerson et al. 2017) that detects CMV‐associated TCR‐β sequences and predicts CMV status from their abundance relative to the total clone count. Across individuals without cancer, the fraction of CMV‐positive (CMV+) individuals increased with age, rising from less than 30% in childhood to more than 65% in older adults (Figure 2f), consistent with an age‐dependent increase in CMV prevalence in the population (Mentzer et al. 2022). Individuals predicted to be CMV+ exhibited consistently lower TCR repertoire diversity than CMV‐negative (CMV‐) individuals, across age and sex (Figure 2g; Table S2). Notably, a CMV+ individual in the 51–60 age group exhibits TCR repertoire diversity comparable to that of a CMV– individual aged 71–80 (segmented regression analysis; Figure S3h,i), suggesting that CMV infection may approximate the effect of two decades of aging on repertoire diversity. Consistent with the effects of age and sex, the impact of CMV on diversity followed a nonlinear pattern, with an increased effect after midlife. Together, these findings indicate that CMV infection imposes an additional reduction in TCR repertoire diversity beyond the effects of age and sex.

### 2.5 TCR Clonal Composition Is Restricted by Age

Beyond repertoire diversity, the TCR landscape can be further characterized by the extent of clonal expansion. To explore this, TCR clones were grouped into four clone size categories based on their proportional contribution to the TCR repertoire: non‐expanded (singletons), low‐expanded (singleton–0.2% of the repertoire), intermediate‐expanded (0.2%–1%), and hyper‐expanded clones (> 1%) (Britanova et al. 2014; Joshi et al. 2020). In individuals without cancer, the proportion of non‐expanded clones consistently declined with age, decreasing from 94.7% to 65.0% across age groups (Figure 3a). In parallel, the fraction of hyper‐expanded clones increased with age, indicating an age‐related shift toward clonal dominance within the immune system. Consistent with the increased rate of decline observed with TCR diversity, this shift accelerated around midlife, with a distinct change in slope between the 41–50 and 51–60 age groups (Figure 3b). In contrast, the proportions of low‐ and intermediate‐expanded clones remained more stable across all age groups. These dynamics of clonal expansion were consistent across the individual cohorts (Figure S4a).

**FIGURE 3:**
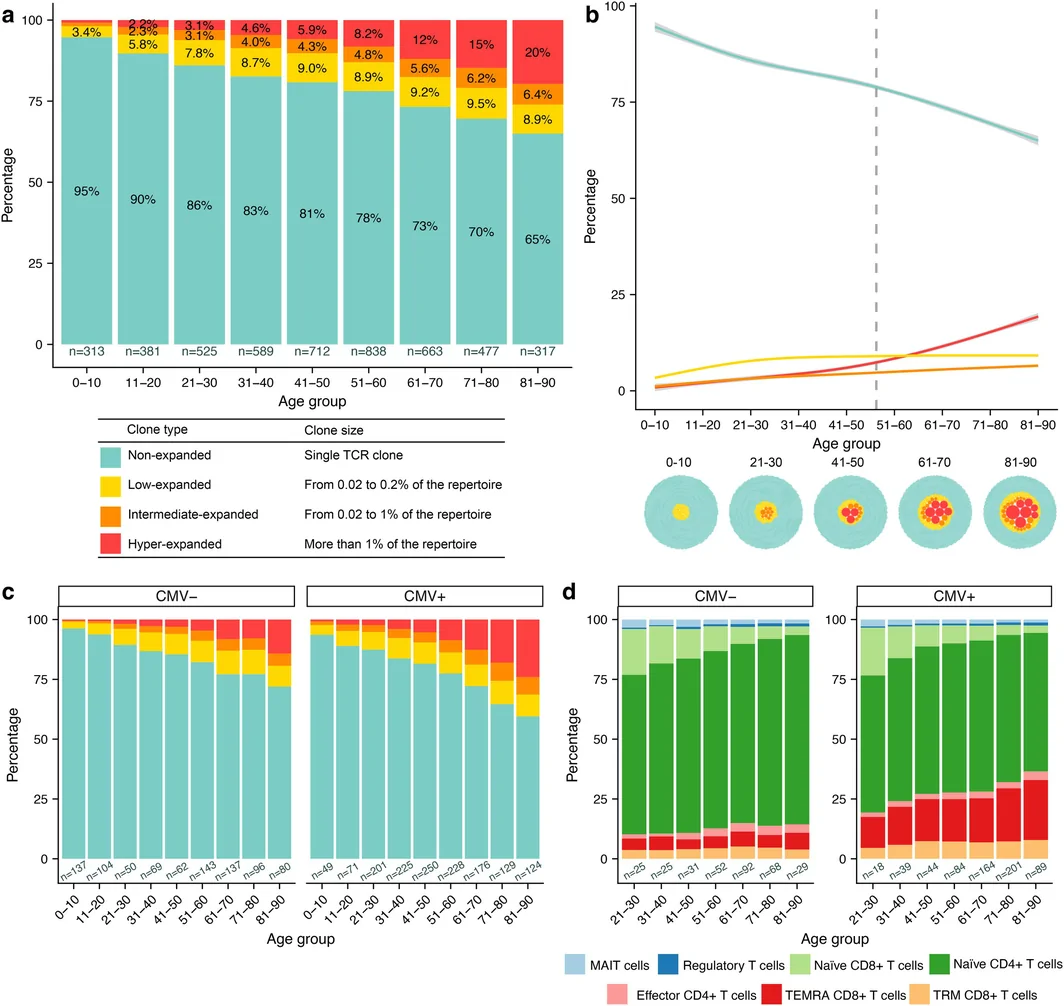
Distribution of TCR clonal composition across age groups. (a) Stacked bar plots showing the relative distribution of T cell clone size categories across age groups in individuals without cancer. (b) Smoothed trends (GAM fits) showing age‐associated changes in the percentage of T cell clone size categories. Shaded areas indicate 95% CIs. The dashed vertical line indicates the estimated breakpoint around age 50. Bubble plots illustrate representative TCR repertoires with median diversity within selected age groups. Each bubble corresponds to a unique TCR clone, with bubble area proportional to clone size and color indicating expansion category. (c) Stacked bar plots showing the relative distribution of T cell clone size categories across age groups in individuals without cancer stratified by CMV status. (d) Stacked bar plots showing the relative distribution of T cell subtypes across age groups in CMV− and CMV+ healthy individuals (OneK1K cohort). MAIT = mucosal‐associated invariant T, TEMRA = T effector memory cells re‐expressing CD45RA, TRM = tissue‐resident memory T. Sample sizes (*n*) for each age group are indicated below the bars. Percentages below 2% are not shown, and percentages above 10% are rounded to the nearest integer.

To investigate how CMV infection influences the clone size distribution, we stratified individuals by predicted CMV status. CMV+ individuals exhibited a higher proportion of hyper‐expanded clones and a lower proportion of non‐expanded clones compared to CMV– individuals, consistent with CMV‐driven clonal expansion (Figure 3c). This enrichment of hyper‐expanded clones was statistically significant across all age groups (Figure S4b). Notably, the shift toward larger clone sizes in CMV+ individuals was evident even in younger age groups, suggesting that a CMV infection can influence the T cell landscape early in life. However, the absolute difference in the proportion of hyper‐expanded clones between CMV+ and CMV– individuals increased with age (Figure S4c), suggesting that CMV‐associated clonal expansion becomes progressively more pronounced in older individuals.

### 2.6 Single‐Cell Sequencing Reveals Age‐ and CMV‐Associated Remodeling of T Cell Subtypes

The T cell landscape comprises several functionally distinct subtypes within both CD4^+^ and CD8^+^ compartments, including naïve, memory, and other populations. However, bulk TCR diversity analyses do not distinguish between individual subtypes. To identify which T cell subtypes change with age and how CMV status influences these changes, we analyzed single‐cell sequencing data of T cells from 981 healthy individuals in the OneK1K (Yazar et al. 2022) cohort. CMV status for each individual was inferred from the single‐cell data using CMVerify (Grabauskas et al. 2026).

We observed a robust age‐associated reduction in naïve CD8^+^ T cells, independent of CMV status (Figure 3d). CMV+ individuals showed a marked enrichment of T Effector Memory re‐expressing CD45RA (TEMRA) CD8^+^ T cells, reflecting a CMV‐driven clonal inflation. Moreover, CMV+ individuals displayed reduced proportions of naïve CD4^+^ T cells relative to CMV– individuals. Together, these patterns indicate that aging primarily depletes the naïve CD8^+^ compartment. Conversely, CMV infection selectively expands cytotoxic effector lineages, yielding a more differentiated T cell landscape in CMV+ individuals.

### 2.7 Patients With Cancer Exhibit Lower TCR Diversity and Greater Clonal Hyper‐Expansion

We have previously observed that reduced T cell diversity and lower T cell abundance are associated with poorer outcomes in patients with bladder cancer (Kjær et al. 2025). We therefore hypothesized that reduced TCR repertoire diversity may represent a broader feature of cancer. To minimize technical variability, we pooled cancer cohorts generated using the same platform (ImmunoSEQ assay: Hamm [cancer subset], Pothuri, Reuben, Snyder and Valpione) and compared them with individuals without cancer of similar chronological age (41–90).

Similar to what we found among individuals without cancer, patients with cancer showed an age‐associated decline in TCR diversity and generally had lower diversity relative to individuals without cancer, although the magnitude of this difference varied across age groups (Figure 4a). The age‐associated decline was also seen within several individual cancer cohorts (Figure S5a). For individuals over 50 years of age, a linear model adjusted for age, sex, and CMV confirmed that cancer status was significantly associated with reduced TCR diversity (estimate = −0.0225, 95% CI: −0.032 to −0.013, *p* < 0.0001; Figure S5b). Based on this model, the average effect of cancer corresponds to approximately 10 years of aging of the TCR repertoire.

**FIGURE 4:**
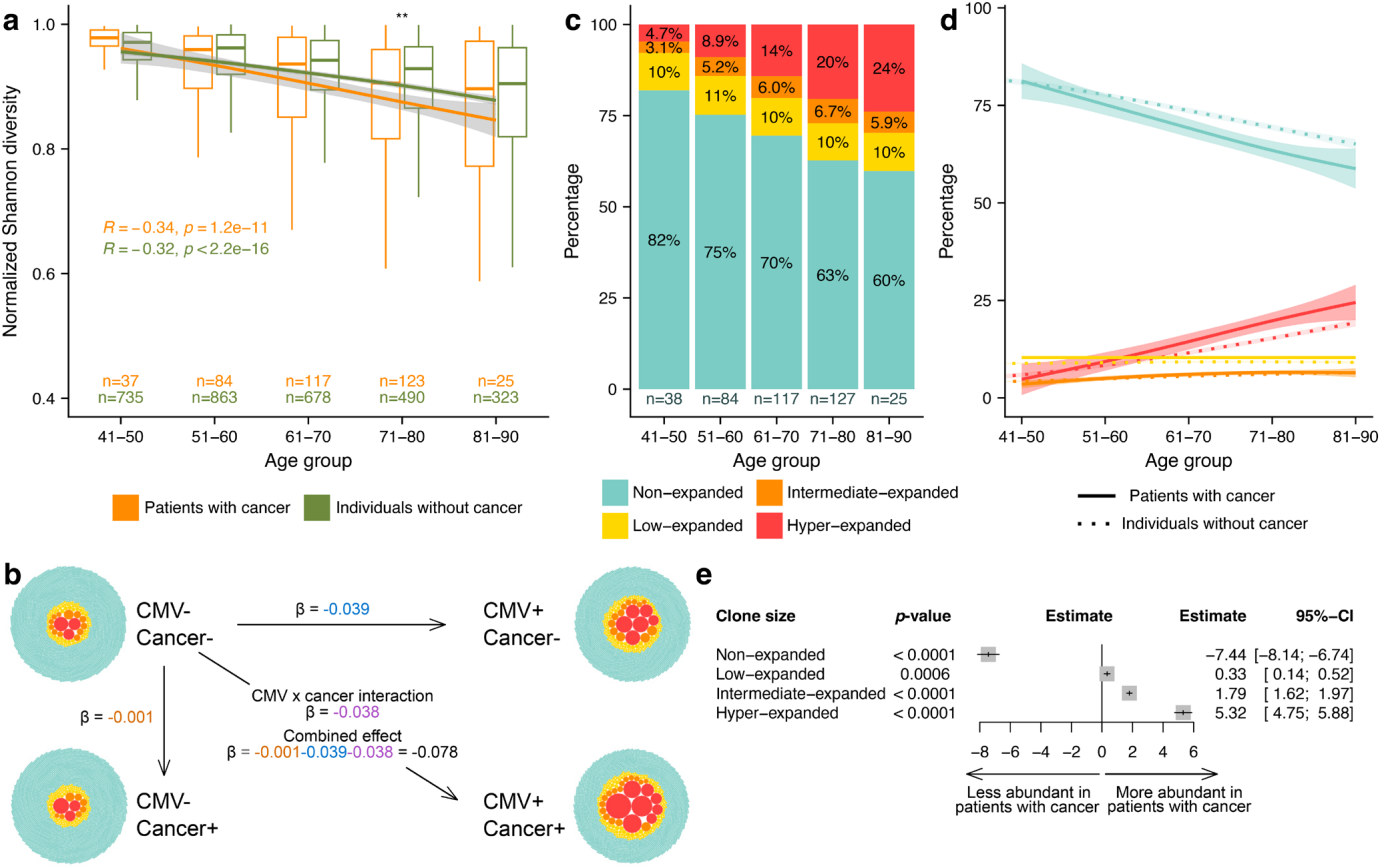
Reduced TCR diversity and altered clonal expansion in patients with cancer. (a) Boxplots show normalized Shannon diversity across 10‐year age groups for patients with cancer and individuals without cancer. Lines represent GAM‐fitted curves with 95% CIs. Spearman correlation coefficients and corresponding *p*‐values are indicated. Sample sizes (*n*) per group are shown below each box. Statistical significance between groups was assessed using the Wilcoxon rank‐sum test (**p < 0.01 ). (b) Schematic illustrating the interaction between CMV and cancer status on normalized Shannon diversity in individuals over 50 years of age. Bubble plots of representative TCR repertoires are shown for CMV− and CMV+ individuals with and without cancer. Each bubble corresponds to a unique TCR clone, with bubble area proportional to clone size and color indicating expansion category. Regression coefficients indicate the estimated effects of CMV and cancer status, together with the CMV × cancer interaction and the combined effect in CMV+ patients with cancer. (c) Stacked bar plots showing the relative distribution of TCR clone size categories across age groups in patients with cancer. Sample sizes (*n*) are shown below each age group. Percentages above 10% are rounded to the nearest integer. (d) GAM‐fitted curves showing age‐associated changes in the proportions of TCR clone size categories in patients with cancer (solid lines) and individuals without cancer (dotted lines). Shaded areas indicate 95% CIs. (e) Forest plot showing the effect of cancer status on the proportion of TCR clones across clone size categories. Estimates were derived from linear models adjusting for age group, sex, and CMV status and are based on cohorts generated using the ImmunoSEQ assay. Negative estimates indicate a lower proportion of clones in patients with cancer, whereas positive estimates indicate a higher proportion in patients with cancer.

As CMV infection and cancer both put strain on the immune system, we asked whether their co‐occurrence leads to a compounded reduction in TCR diversity. Indeed, we found that the effect of CMV infection on TCR diversity was more pronounced in patients with cancer (interaction model, *p* = 0.0001; Figure 4b; Figure S5c). Notably, among CMV− individuals, no association between cancer and TCR diversity was observed, suggesting that the reduced diversity seen in patients with cancer is primarily driven by an amplification of the CMV effect, potentially reflecting reduced immunological control of CMV in the context of cancer.

To determine whether cancer is associated with shifts in TCR clone size distributions, we compared the relative proportions of hyper‐expanded, intermediate‐expanded, low‐expanded, and non‐expanded clones. Patients with cancer showed an age‐associated shift in clonal composition similar to that observed in individuals without cancer, characterized by depletion of non‐expanded clones and enrichment of hyper‐expanded clones (Figure 4c). This pattern was consistent across cohorts, although we observed some assay‐specific shifts in clonal composition (Figure S5d). Interestingly, the shift was more pronounced in patients with cancer than in individuals without cancer (Figure 4d). Although these differences were not statistically significant within all age groups (Table S3), linear modeling showed that cancer status was associated with differences across all clone size categories after accounting for age, sex and CMV status (Figure 4e). The most pronounced differences were a reduced proportion of non‐expanded clones (estimate = −7.44, 95% CI: −8.14 to −6.74, *p* < 0.0001) and an increased proportion of hyper‐expanded clones (estimate = 5.32, 95% CI: 4.75–5.88, *p* < 0.0001). Consistent with the findings for TCR diversity, cancer‐associated differences in clonal composition were most pronounced among CMV+ individuals (Figure S6). Together, these results indicate that cancer is associated with a more contracted and clonally skewed TCR repertoire, resembling that of individuals of older chronological age.

### 2.8 High TCR Repertoire Diversity Is Associated With Improved Cancer Outcome

Because TCR diversity reflects the breadth of the circulating T cell compartment, we asked whether variation in diversity carries prognostic significance in cancer. To assess associations with overall survival (OS), we performed Cox proportional hazards regression across seven cancer cohorts (pancreatic, lung, bladder, breast, melanoma, and urothelial cancers), with hazard ratios estimated per 0.1‐unit increase in TCR diversity. Higher TCR repertoire diversity was generally associated with improved OS (hazard ratio [HR] = 0.86, 95% CI: 0.77–0.96, *p* = 0.0078; Figure 5a). Although the effect size varied between cohorts, all HRs were below 1, with the strongest association observed in the Snyder cohort (HR = 0.52, 95% CI: 0.29–0.92, *p* = 0.03; Figure 5a). Together, these results indicate a trend toward better survival among patients with higher TCR diversity. Given the pronounced association between CMV status and TCR diversity, we examined whether CMV itself was associated with survival, and found that CMV status was not significantly associated with OS (Figure S7).

**FIGURE 5:**
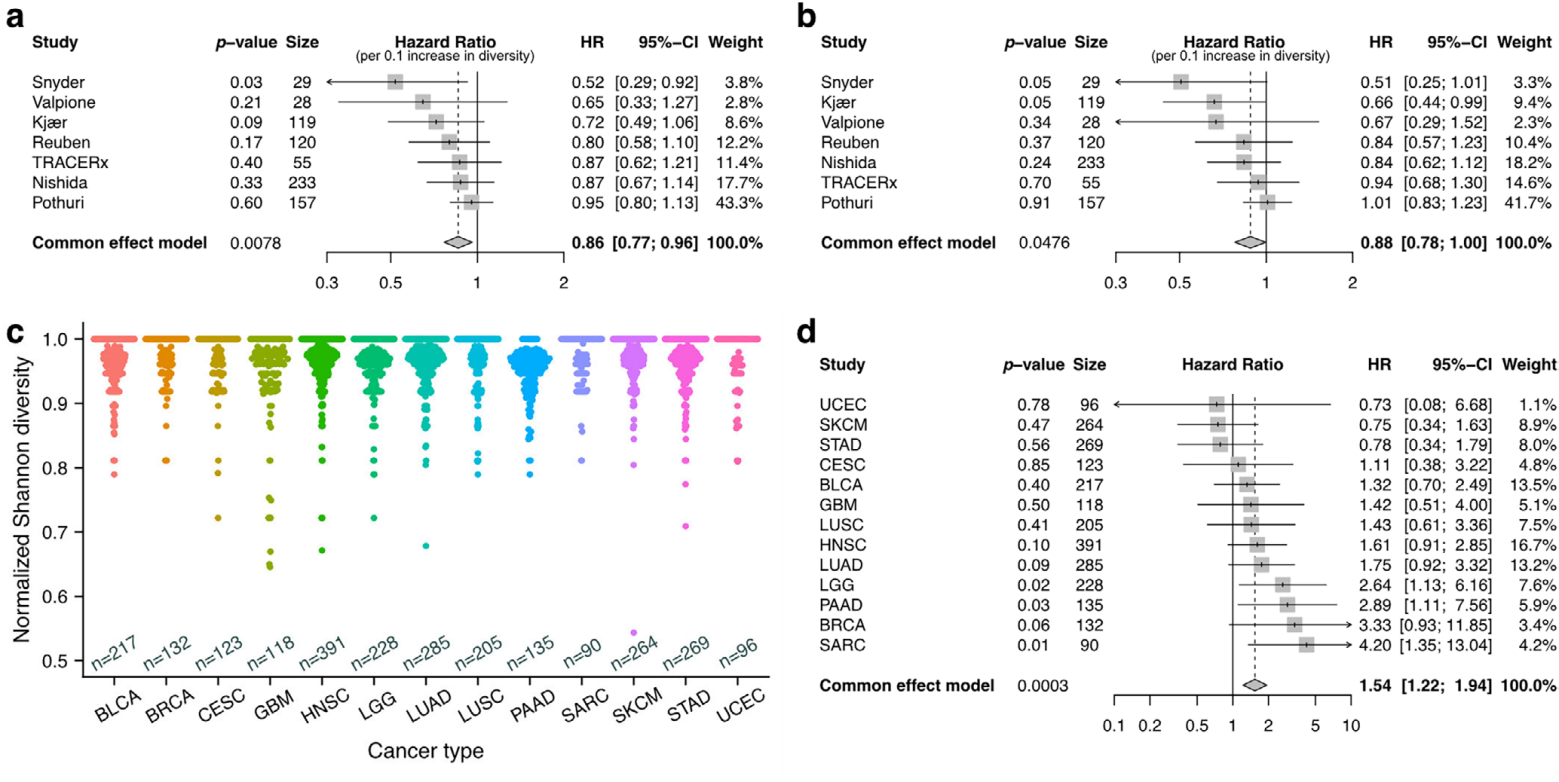
Association of TCR repertoire diversity with cancer outcomes. (a) Forest plot summarizing Cox proportional hazards regression analyses of the association between normalized Shannon diversity and OS across seven independent cancer cohorts. Hazard ratios represent the effect per 0.1 increase in normalized Shannon diversity. (b) Forest plot summarizing Cox proportional hazards regression analyses of the association between age, sex, and CMV−corrected TCR repertoire diversity and OS. Hazard ratios represent the effect per 0.1 increase in corrected diversity. (c) Distribution of normalized Shannon diversity across TCGA cancer types, with sample sizes (*n*) indicated below each cancer type. (d) Forest plot summarizing Cox proportional hazards regression analyses across TCGA cancer types comparing patients with both low TCR diversity and low T‐cell abundance to all other patients, adjusted for age and sex.

Because age, sex, and CMV status substantially influence peripheral TCR diversity, we next asked whether deviations from the diversity expected based on these host factors were associated with survival. Using the linear model trained on individuals without cancer (Figure S3i), we estimated the TCR diversity expected for each patient with cancer based on their age, sex, and CMV status. We then calculated the difference between the observed and expected diversity (observed minus predicted), providing a measure of whether an individual's TCR repertoire was more or less diverse than expected given these characteristics. A positive value therefore represents a more diverse TCR repertoire than expected, potentially reflecting a biologically younger immune profile relative to that expected based on the given host factors, whereas a negative value represents a less diverse repertoire, potentially reflecting a biologically older immune profile. We subsequently tested whether deviations from the expected TCR diversity were associated with OS using Cox proportional hazards regression. Higher than expected TCR diversity was significantly associated with improved OS (Figure 5b), suggesting that a biologically younger TCR repertoire, relative to that expected based on age, sex, and CMV status, is associated with more favorable outcomes.

### 2.9 Low TCR Repertoire Diversity and Low T Cell Fraction Are Linked to Worse Survival in TCGA

To further explore the association between TCR diversity and survival in an independent cohort, we collected peripheral blood WES data from TCGA and estimated the TCR repertoire diversity using MiXCR's (Bolotin et al. 2017, 2015) framework for fragmented shotgun exome sequencing data (Figure 5c). Due to the limited number of TCR reads in this dataset (3–57 TCR reads pr. sample), diversity estimates at the individual sample level were relatively noisy. To obtain more stable and interpretable comparisons within each cancer type, patients were stratified into low (bottom quartile) and high (above the bottom quartile) diversity groups. A multivariable Cox model across cancer types, adjusted for age and sex, showed that patients with low TCR diversity had significantly worse OS (HR = 1.21, 95% CI: 1.06–1.39; *p* = 0.0051; Figure S8a).

While TCR diversity reflects the evenness and breadth of the clonal repertoire, it does not capture the overall quantity of circulating T cells. To determine whether total T cell abundance also carried prognostic value, we estimated T cell fractions from WES data using T cell ExTRECT (Bentham et al. 2021) and stratified patients within each cancer type into low (bottom quartile) and high (above the bottom quartile) groups. Meta‐analysis demonstrated a significant association between low T cell fractions and worse OS (HR = 1.18, 95% CI: 1.05–1.33, *p* = 0.0043; Figure S8b).

As TCR diversity and T cell abundance provide complementary information about the peripheral T cell compartment, we next assessed their combined prognostic value. Across three cancer types, patients with both low diversity and low T cell abundance experienced significantly worse OS (Figure 5d). A meta‐analysis across all cancer types yielded an overall HR of 1.54 (95% CI: 1.22–1.94, *p* = 0.0003; Figure 5d). Notably, the effect size varied between cancer types, suggesting that the prognostic relevance of TCR diversity and T cell abundance may be cancer‐type specific.

## 3 Discussion

In this study, we investigated how age, sex, and CMV infection shape peripheral blood TCR repertoire diversity across the human lifespan and in cancer. By integrating data from multiple cohorts, we identified a nonlinear decline in TCR diversity that accelerates after midlife and is modified by sex and CMV status. We further observed that variation in TCR diversity was associated with OS across seven cancer cohorts.

Consistent with previous studies, we observed a gradual decline in TCR repertoire diversity with advancing age (Weng 2023; Yoshida et al. 2017; Zahid et al. 2024). A central finding of our analysis is that age‐associated loss of TCR diversity is not constant throughout adulthood, but accelerates after approximately 50 years of age. Segmented regression analysis revealed a marked increase in the rate of decline beyond this point, consistent across multiple diversity metrics. This observation advances our understanding of TCR repertoire dynamics and highlights a critical transition point in immune aging.

Furthermore, we observed an age‐dependent shift in overall clonal composition, characterized by a decline in the fraction of non‐expanded T cells and a corresponding increase in hyper‐expanded clones. The reduction in non‐expanded clones may reflect decreased production of naïve T cells due to age‐associated thymic involution (Palmer 2013), while the accumulation of hyper‐expanded clones may reflect repeated antigen exposure over the lifespan. Single‐cell analyes from the OneK1K cohort indicated that this age‐related shift was driven predominantly by a loss of naïve CD8^+^ T cells.

We further showed that sex contributes independently to variation in TCR diversity. Consistent with previous reports (Trofimov et al. 2022; Zahid et al. 2024), males exhibited lower repertoire diversity than females across age groups. This finding is supported by studies of immune aging showing pronounced sexual dimorphism in immune system remodeling, including accelerated immune aging and reduced thymic output in males compared with females (Márquez et al. 2020). This difference may reflect sex‐specific rates of thymic involution, with females exhibiting a delayed onset and slower progression than males, thereby maintaining a more diverse TCR repertoire, as reported previously (Araki et al. 2016; Cardinale et al. 2021). These results underscore the importance of accounting for sex as a biological variable when studying immune aging.

CMV status was shown to be highly associated with TCR repertoire diversity, with CMV+ individuals exhibiting consistently lower diversity across the lifespan. This supports previous findings that chronic CMV infection drives memory inflation through persistent antigen exposure, leading to clonal expansion and narrowing of the overall repertoire (Zhuo et al. 2022). We found that CMV+ individuals exhibited repertoire diversity comparable to that of CMV− individuals approximately two decades older. However, despite this marked reduction in diversity, CMV status was not associated with OS across the cancer cohorts analyzed. This observation is consistent with several previous studies reporting none or only modest effects of CMV positivity on all‐cause mortality (Chen et al. 2021; Doorly et al. 2025; Huang et al. 2024; Matheï et al. 2015). Notably, CMV positivity has been associated with increased all‐cause mortality, corresponding to an estimated reduction in life expectancy of approximately 3.7 years (Savva et al. 2013). This reported survival impact of CMV is modest relative to the pronounced impact of CMV on TCR repertoire diversity observed in this study. Thus, although CMV affects TCR repertoire diversity, this remodeling of the TCR repertoire has limited effect on survival. We hypothesize that CMV alters the relative clonal composition of the T cell pool without causing a broad loss of immune competence. Supporting this, previous reports have shown that expansion of CMV‐specific clones does not decrease the diversity of the remaining repertoire (Lindau et al. 2019). In the context of T cell subtypes, CMV was associated with an expansion of TEMRA CD8^+^ T cells, which simultaneously compressed the relative fraction of naïve CD4^+^ T cells. Importantly, this does not necessarily imply a depletion of naïve CD4^+^ T cells (Lindau et al. 2019). In contrast, aging was associated with a decline in naïve CD8^+^ T cells, while CMV had little effect on this population. Together, these observations suggest that CMV‐driven immune remodeling differs from immune aging. CMV primarily alters the relative repertoire composition through expansion of TEMRA CD8^+^ T cells, whereas aging is associated with the loss of new naïve T cells and a consequent reduction in immune competence. The consequence of this CMV‐associated remodeling is likely to be disease and treatment‐specific. In our cohorts, treated primarily with chemotherapy, we find no association between CMV and survival. Conversely, it has recently been reported that CMV status was associated with improved outcomes in patients treated with anti‐PD‐1 immunotherapy (Milotay et al. 2025), indicating that CMV‐associated immune remodeling might be advantageous in some settings. This effect could be mediated through bystander activation of CMV‐specific TEMRA CD8^+^ T cells (Ghirardelli Smith et al. 2026), however, further research is needed to uncover a potential mechanism. Notably, we observed that the CMV‐associated remodeling appeared to be even more pronounced in patients with cancer, indicating that CMV and cancer might jointly shape T cell remodeling. Collectively, our results underscore the importance of accounting for CMV status as a confounder and key factor in future investigations of immunosenescence and age‐related immune decline.

In the context of cancer survival, we found that peripheral TCR repertoire diversity was associated with improved OS across multiple cancer types. This association was also observed when considering deviations from the diversity expected based on age, sex, and CMV status, as estimated using a regression model trained in individuals without cancer. Analysis of the independent TCGA cohort further showed that the combination of low TCR diversity and low T cell abundance identified patients with particularly poor outcomes. These findings align with previous reports linking reduced repertoire diversity and diminished T cell abundance to adverse disease outcomes (Bentham et al. 2025; Charles et al. 2020; Kjær et al. 2025), suggesting the potential use of T cell measures as informative biomarkers for risk stratification and prognosis. Importantly, these immune characteristics can be assessed non‐invasively from peripheral blood, suggesting potential utility as a complement to existing clinical and molecular prognostic indicators. Such measures may help identify patients who could benefit from closer monitoring. However, we also observed variability in the strength of association across cancer types. This heterogeneity suggests that the prognostic relevance of peripheral TCR diversity and T cell abundance may be cancer type specific. Future studies should aim to clarify these differences and determine the settings in which peripheral immune features provide the greatest prognostic value. A limitation of this study is the heterogeneity of the included cancer cohorts with respect to treatment history, treatment type, sampling timing, and disease stage. Although most cancer cohorts included pretreatment samples, some cohorts included patients who had received prior systemic treatment. Because treatment can alter the peripheral T‐cell repertoire and also influence OS, treatment‐related differences may confound the observed association between TCR diversity and survival. Likewise, variability in sampling timing relative to treatment initiation may have contributed to differences in peripheral TCR diversity across cohorts.

In summary, this study establishes peripheral TCR repertoire diversity as a dynamic marker of immune aging shaped by both intrinsic factors, including age and sex, and extrinsic influences such as chronic CMV infection. We identify a nonlinear trajectory of TCR diversity loss that accelerates after midlife and is further modified by sex and CMV status. This highlights how these factors jointly influence immune competence across the human lifespan. Importantly, we show that variation in TCR diversity carries prognostic relevance across multiple cancer types, even when accounting for the effects of major demographic and viral confounders on diversity. Together, these findings underscore the importance of accounting for chronic viral exposures in studies of immunosenescence. Furthermore, it supports the use of immune repertoire features as scalable biomarkers of immune health and disease outcome, with potential to enhance prognostic accuracy and inform personalized treatment strategies.

## 4 Methods

### 4.1 Datasets and Preprocessing

Ten datasets (Mitchell, Emerson, Delmonte, Nolan, Gittelman, Hamm, Pothuri, Reuben, Snyder and Valpione) were retrieved from the immuneAccess database managed by Adaptive Biotechnology. Datasets collected from immuneAccess were processed using the ImmunoSEQ assay. ZIP files were downloaded using the export function with version set to v2. The remaining datasets (Britanova et al. 2016; Joshi et al. 2020; Kjær et al. 2025; Nishida et al. 2023), were downloaded from literature‐provided repositories. Detailed information for each dataset is provided in Table S1. Individuals older than 90 years were excluded from all primary analyses due to limited sample representation in this age range. For cohorts with repeated sampling, only the first sample per individual was retained. In the Mitchell cohort, additional samples were included if collected at least 2 years after the most recently retained sample. Among the cancer cohorts, samples from the TRACERx, Nishida, Reuben, Kjær, and Valpione cohorts were collected prior to treatment. In the Snyder cohort, samples were collected before initiation of immune checkpoint inhibitor therapy, although patients had received prior systemic treatment. In the Pothuri cohort, samples were collected during surgical resection, and patients had received chemotherapy, chemoradiation therapy, or no neoadjuvant therapy. Nonproductive CDR3 sequences were excluded to ensure that each TCR sequence represented a functional clone. To improve comparability across individuals, we minimized differences between cohorts by randomly downsampling all samples. To select an appropriate downsampling depth that balances diversity preservation with sample inclusion, we evaluated how sample size affected TCR repertoire diversity (Figure S1). Based on this analysis, we downsampled all samples to 5000 randomly selected TCR‐β sequences, a threshold that preserves diversity while minimizing the exclusion of individuals with low T cell counts. For cohorts generated using unique molecular identifiers (UMIs), samples with fewer than 5000 TCR‐β clones were excluded. The number of excluded samples for each cohort is summarized in Table S4. In contrast, no samples were excluded from the Kjær cohort, which was generated without UMIs and exceeded this threshold in all cases. The normalized Shannon diversity index was calculated using the formula:

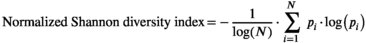

where *N* represents the total number of unique clones present in the sample, and $p_{i}$ represents the frequency of clone i. The Simpson diversity index was calculated as:

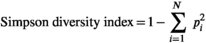

The Gini index was calculated using the repDiversity function from the immunarch R package (Popov et al. 2025). The Gini coefficient is defined as:

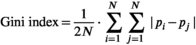




### 4.2 CMV Status Prediction

To infer CMV status, we applied a predictive model based on the presence of CMV‐associated TCR‐β clones in the peripheral blood. A previously published list (Emerson et al. 2017) of CMV‐associated TCR‐β sequences was used to quantify the number of CMV‐associated clones in each sample. A logistic regression model was then applied to predict the probability of CMV positivity based on the number of CMV‐associated TCR‐β sequences and the number of unique TCR‐β clones for each individual. Predicted CMV probabilities were converted to binary CMV status using a probability threshold of 0.5. The model was trained and validated on the Emerson cohort, which included 641 individuals in the training set and 120 in the validation set, all with known CMV status. The classifier demonstrated high accuracy, achieving an area under the curve (AUC) of 0.98 on the training set and 0.94 on the validation set (Figure S9), effectively distinguishing CMV+ from CMV− individuals.

### 4.3 Auxiliary Data

TCGA WES BAM files derived from peripheral blood samples were downloaded from the GDC Data Portal, and productive CDR3 sequences from the TCR β‐chain were extracted using MiXCR's (Bolotin et al. 2017, 2015) (v3.0.13) analyze shotgun functionality. In addition, patients with fewer than three reads spanning the TCR or fewer than two unique clones were excluded from the analysis, as diversity could not be reliably estimated in these cases; such samples yield a fixed diversity value of 1, regardless of the underlying repertoire composition. T cell ExTRECT‐based T cell fraction estimates from peripheral blood were obtained from (Bentham et al. 2025). Patients with missing age information were excluded. For analysis of TCR diversity in TCGA, cancer types represented by fewer than 50 patients with sufficient TCR reads were excluded from analysis. For T cell ExTRECT analysis, cancers with fewer than 50 patients with T cell fraction estimates were excluded. In addition, cancer types that had fewer than 10 death events were excluded to ensure stable estimations in the survival analysis.

### 4.4 Single‐Cell Analyses

Single‐cell RNA sequencing count data from 981 healthy individuals were obtained from the OneK1K cohort (Yazar et al. 2022). Cell type annotations generated using CellTypist (Xu et al. 2023) were used to quantify the relative abundance of major T cell subtypes across age groups. CMV status for each individual was inferred from single‐cell TCR‐seq data using the CMVerify (Grabauskas et al. 2026) framework.

### 4.5 Modeling Age‐, Sex‐, and CMV‐Associated Effects on TCR Diversity

To assess how age‐related changes in TCR repertoire diversity differ after midlife and whether these effects are modified by sex and CMV status, we fitted linear regression models including interaction terms between age (modeled per 10‐year group) and a binary indicator for individuals older than 50 years. Sex and predicted CMV status were included as additional covariates, each with a corresponding interaction term with the binary age‐indicator. Effects were estimated separately for individuals ≤ 50 years and > 50 years using estimated marginal trends and contrasts implemented in the emmeans (Lenth 2025) R package. For individuals > 50 years, reported estimates reflect the combined (main and interaction) effects and were used for visualization in forest plots. *p* values shown in the forest plots correspond to these combined contrasts. Notably, all interaction terms capturing age‐dependent modifications of the effects were statistically significant.

### 4.6 Age, Sex, and CMV Correction of TCR Repertoire Diversity

To isolate variation in TCR repertoire diversity independent of demographic and viral influences, we derived age‐, sex‐, and CMV‐corrected diversity estimates using the linear regression framework described above. Models were fitted in individuals without cancer to estimate expected normalized Shannon diversity as a function of age, sex, and predicted CMV status. For the Valpione cohort, which lacked sex information, a corresponding model excluding sex was used to maximize sample inclusion in the analysis. For each cancer sample, corrected diversity was defined as the residual difference between observed diversity and the value predicted by the model.

### 4.7 Statistical Analysis

Statistical analyses were performed using R (v4.4.2). Data visualization was primarily performed using the ggplot2 (Wickham 2009) R package, and multi‐panel figures were assembled using the cowplot (Wilke 2025) R package. All pairwise comparisons were assessed using the Wilcoxon rank‐sum test (two‐sided), with *p*‐values visualized using the ggpubr (Kassambara 2025) R package. For boxplots, the center line represents the median, the box represents the interquartile range (IQR), and the whiskers extend to the furthest data points within 1.5 × IQR. For forest plots, the center of the gray box represents the estimated value, while the whiskers represent the 95% CI of the estimate. For survival analyses, HRs, two‐sided *p*‐values, and 95% CIs were estimated using Cox proportional hazards regression implemented in the R package survival (Terry and Therneau 2000; Therneau 2024). When TCR repertoire diversity was modeled as a continuous variable, HRs represent the relative change in risk per 0.1 increase in normalized Shannon diversity. Forest plots and meta‐analyses across cohorts were performed using the R package meta (Balduzzi et al. 2019). Receiver operating characteristic (ROC) curves and AUC values were generated using the R package pROC (Robin et al. 2011). Scatter plots with quasi‐randomized point distribution were generated using the R package ggbeeswarm (Clarke et al. 2025). Trend lines were fitted using either linear models (LMs) or generalized additive models (GAMs) implemented with cubic regression splines and shrinkage (bs = “cs”), with the number of basis functions restricted to *k* = 5 to limit model complexity and avoid overfitting, using the geom_smooth function in ggplot2. GAMs were initially used to explore relationships without assuming a linear association, allowing potential non‐linear patterns in the data to be identified. Where the fitted GAM curves suggested a change in slope, separate linear models were subsequently fitted within the approximately linear regions before and after the apparent change in slope to provide simpler and more interpretable slope estimates of the observed relationships. Breakpoint analysis of age‐associated changes in TCR diversity was performed using segmented linear regression implemented in the R package segmented (Muggeo 2003, 2008). Correlations were assessed using Spearman's rank correlation, and derivatives were calculated using the R package gratia (Simpson 2024, 2025). Throughout, *p*‐values below 0.05 were considered statistically significant. No adjustments for multiple testing were applied unless otherwise stated. All statistical tests performed were two‐sided unless otherwise stated.

## Author Contributions

N.L., A.K.A., and R.I.J. contributed equally. N.L. and A.K.A. gathered the datasets. N.L., A.K.A., and R.I.J. performed analyses. N.L. wrote the statistical code. N.L., A.K.A., R.I.J., and N.J.B. drafted the manuscript. N.J.B. supervised the study. All authors provided feedback and interpretation of results. All authors approved the final version of the manuscript.

## Funding

This work was supported by the Lundbeck Foundation (R272‐2017‐4040), the Novo Nordisk Foundation (NNF21OC0071483 and NNF23OC0085954), and a Savvaerksejer Jeppe Juhl og Hustru Ovita Juhl research stipend.

## Conflicts of Interest

N.M. holds patents related to determining human leukocyte antigen (HLA) LOH (PCT/GB2018/052004), determination of B cell fraction in mixed samples (PCT/EP2024/062999), determination of lymphocyte abundance in mixed samples (PCT/EP2022/070694), identifying responders to cancer treatment (PCT/GB2018/051912), targeting neoantigens (PCT/EP2016/059401), identifying patient response to immune checkpoint blockade (PCT/EP2016/071471) and predicting survival rates of patients with cancer (PCT/GB2020/050221) and has a patent pending in determining HLA disruption (PCT/EP2023/059039). H.J.W.L.A. reports consulting fees and/or stock from Onc.AI, Love Health, Sphera, Health‐AI, Ambient, and AstraZeneca. L.D. has sponsored research agreements with C2i Genomics, Natera, AstraZeneca, Photocure, and Ferring and has an advisory/consulting role at Ferring, MSD, Cystotech and UroGen. L.D. has received speaker honoraria from AstraZeneca, Pfizer, and Roche and travel support from MSD. L.D. is a board member at BioXpedia. ADF reports fees and advisory fees from Novartis, IQVIA, Hanson‐Wade. N.J.B. reports consulting fees from Ambient, is listed as a co‐inventor on a patent application (PCT/GB2020/050221) on methods for cancer prognostication and a patent on methods for predicting anti‐cancer response (US14/466,208). None of the other authors have any conflicts to declare.

## Supporting information


**Figure S1:** Subsampling analysis of TCR repertoire diversity. Downsampling curves showing the effect of sampling depth on TCR repertoire diversity in the Hamm cohort. Each line represents an individual sample.
**Figure S2:** Sex‐specific trajectories of age‐associated TCR repertoire diversity across cohorts. Normalized Shannon diversity as a function of age is shown for each cohort comprising individuals without cancer, with GAM fits and 95% CIs stratified by sex (female, pink; male, blue). Spearman correlation coefficients and corresponding *p*‐values are indicated, and sample sizes (*n*) are provided for each cohort.
**Figure S3:** Age‐associated acceleration of TCR repertoire diversity loss. (a) Forest plot showing effect estimates for the per–10‐year age‐group decline in normalized Shannon diversity in individuals ≤ 50 years and > 50 years. (b, c) Quasirandom scatter plots showing age‐associated changes in (b) Simpson index and (c) Gini coefficient across 10‐year age groups. Red lines indicate GAM fits with 95% CIs. (d, e) Forest plot showing effect estimates for the per–10‐year age‐group change in (d) Simpson index and (e) Gini coefficient in individuals ≤ 50 years and > 50 years. (f) Age‐associated decline in normalized Shannon diversity shown separately for individuals ≤ 50 years and > 50 years, stratified by sex. Lines indicate linear fits with 95% CIs. (g) Forest plot showing effect estimates for age‐group and sex‐associated changes in normalized Shannon diversity in individuals ≤ 50 years and > 50 years. (h) Age‐associated decline in normalized Shannon diversity shown separately for individuals ≤ 50 years and > 50 years, stratified by sex and CMV status, with linear fits and 95% CIs. (i) Forest plot showing effect estimates for age‐group‐, sex‐, and CMV‐associated changes in normalized Shannon diversity in individuals ≤ 50 years and > 50 years.
**Figure S4:** Age‐associated shifts in TCR clonal expansion across cohorts. (a) Stacked bar plots showing the relative distribution of T cell clone size categories across age groups within each cohort comprising individuals without cancer. (b) Boxplots showing the percentage of hyper‐expanded clones across age groups, stratified by CMV status, based on the cohorts comprising individuals without cancer generated using the ImmunoSEQ assay. *p*‐values were calculated using the Wilcoxon rank‐sum test (****p < 0.0001). (c) Absolute difference in the proportion of hyper‐expanded clones between CMV+ and CMV− individuals across age groups, based on the cohorts comprising individuals without cancer generated using the ImmunoSEQ assay.
**Figure S5:** Age‐, sex‐, CMV‐ and cancer‐associated changes in TCR repertoire diversity and clonal expansion. (a) Normalized Shannon diversity as a function of age within each cancer cohort. GAM fits with 95% confidence intervals are shown, stratified by sex (female, pink; male, blue; unknown, gray). Spearman correlation coefficients and corresponding *p*‐values are indicated, and sample sizes are provided for each cohort. (b) Forest plot showing regression coefficients from a linear model fitted to individuals over 50 years of age. The plot summarizes associations between age group, sex, CMV status, cancer status, and normalized Shannon diversity. (c) Forest plot showing regression coefficients from a linear model fitted to individuals over 50 years of age, including an interaction between CMV and cancer status and adjustment for age group and sex. Analyses in (b) and (c) were restricted to cohorts generated using the ImmunoSEQ assay. Regression coefficients represent the estimated difference in normalized Shannon diversity for the category indicated in brackets relative to its reference category. The age‐group coefficient represents the estimated change in normalized Shannon diversity per 10 years, while the interaction coefficient represents the additional association between CMV positivity and cancer status beyond their individual associations. (d) Stacked bar plots showing the relative distribution of T cell clone size categories across age groups within each cancer cohort. Sample sizes are indicated below each bar.
**Figure S6:** Associations of age, sex, CMV status, cancer status, and the interaction between CMV and cancer status with clonal size distribution. Forest plots showing regression coefficients from linear models for the percentage of (a) hyper‐expanded, (b) intermediate‐expanded, (c) low‐expanded, and (d) non‐expanded TCR clones. Models included age group, sex, CMV status, cancer status, and an interaction between CMV and cancer status. Regression coefficients represent estimated changes in percentage points. For categorical predictors, estimates are relative to the corresponding reference categories, while the interaction coefficient represents the additional association between CMV positivity and cancer status.
**Figure S7:** Prognostic impact of CMV status on overall survival. Forest plot summarizing Cox proportional hazards regression analyses assessing the association between CMV status and OS across independent cancer cohorts. Hazard ratios are shown for CMV+ individuals relative to CMV− individuals.
**Figure S8:** Prognostic impact of TCR diversity and T cell fraction across TCGA cancer types. (a–b) Forest plots summarizing Cox proportional hazards regression analyses adjusted for age and sex, assessing the association between OS and (a) normalized TCR repertoire diversity groups and (b) T cell fraction groups across TCGA cancer types. Groups were defined using the 25th percentile threshold (bottom 25% vs. top 75%), with the top 75% serving as the reference group.
**Figure S9:** CMV status classification model. (a) Scatter plot showing the relationship between the number of unique TCR‐β clones and CMV‐associated TCR‐β clones, stratified by CMV status in the Emerson cohort. (b) Receiver operating characteristic (ROC) curves illustrating classifier performance in distinguishing CMV+ and CMV− individuals in the training and validation cohorts.
**Table S1:** Overview of cohorts included in this study. Summary of all cohorts used for TCR repertoire analyses, including sample size, disease context, input material, sequencing method and sample timing.
**Table S2:** Comparison of TCR repertoire diversity by CMV status across age groups and sexes. Wilcoxon rank‐sum test *p*‐values comparing normalized Shannon diversity between individuals predicted to be CMV+ and CMV− within each age group, stratified by sex.
**Table S3:** Cancer‐associated differences in TCR clone size distributions across age groups. Wilcoxon rank‐sum test *p*‐values comparing the proportions of non‐expanded and hyper‐expanded TCR clones between patients with cancer and individuals without cancer within each age group.
**Table S4:** Cohort‐specific sample exclusions following downsampling. Samples generated using UMI‐based sequencing were excluded if they contained fewer than 5000 TCR‐β clones. The table summarizes the number of excluded samples in each cohort.

## Data Availability

All TCR sequencing datasets used in this study are publicly available and were downloaded from the respective publications. TGCA data is available for research use through the NCI Genomic Data Commons (GDC) portal. Onek1k single‐cell count matrices were downloaded from CZ Cellxgene data portal.
