# Supplementary material for: Nonlinear Aging of the Human TCR Repertoire Is Influenced by Sex, CMV, and Cancer
Source: Aging Cell. 2026 Oct 25;25(11):e70777. doi: 10.1111/acel.70777 (PMC13696440; doi:10.1111/acel.70777)
Supplement: Supplementary file 1 [file ACEL-25-e70777-s001.docx]

**Supplementary materials:**

**Figure S1:** Subsampling analysis of TCR repertoire diversity.

**Figure S2:** Sex-specific trajectories of age-associated TCR repertoire diversity across cohorts.

**Figure S3:** Age-associated acceleration of TCR repertoire diversity loss.

**Figure S4:** Age-associated shifts in TCR clonal expansion across cohorts.

**Figure S5:** Age-, sex-, CMV- and cancer-associated changes in TCR repertoire diversity and clonal expansion.

**Figure S6:** Associations of age, sex, CMV status, cancer status, and the interaction between CMV and cancer status with clonal size distribution.

**Figure S7:** Prognostic impact of CMV status on overall survival.

**Figure S8:** Prognostic impact of TCR diversity and T cell fraction across TCGA cancer types.

**Figure S9:** CMV status classification model.

**Table S1:** Overview of cohorts included in this study.

**Table S2:** Comparison of TCR repertoire diversity by CMV status across age groups and sexes.

**Table S3:** Cancer-associated differences in TCR clone size distributions across age groups.

**Table S4:** Cohort-specific sample exclusions following downsampling.

**Figure S1:** Subsampling analysis of TCR repertoire diversity**.** Downsampling curves showing the effect of sampling depth on TCR repertoire diversity in the Hamm cohort. Each line represents an individual sample.

**Figure S2:** Sex-specific trajectories of age-associated TCR repertoire diversity across cohorts**.** Normalized Shannon diversity as a function of age is shown for each cohort comprising individuals without cancer, with GAM fits and 95% CIs stratified by sex (female, pink; male, blue). Spearman correlation coefficients and corresponding p-values are indicated, and sample sizes (n) are provided for each cohort.

**Figure S3:** Age-associated acceleration of TCR repertoire diversity loss. **(a)** Forest plot showing effect estimates for the per–10-year age-group decline in normalized Shannon diversity in individuals ≤ 50 years and > 50 years. **(b, c)** Quasirandom scatter plots showing age-associated changes in (b) Simpson index and (c) Gini coefficient across 10-year age groups. Red lines indicate GAM fits with 95% CIs. **(d, e)** Forest plot showing effect estimates for the per–10-year age-group change in (d) Simpson index and (e) Gini coefficient in individuals ≤ 50 years and >50 years. **(f)** Age-associated decline in normalized Shannon diversity shown separately for individuals ≤50 years and > 50 years, stratified by sex. Lines indicate linear fits with 95% CIs. **(g)** Forest plot showing effect estimates for age-group and sex-associated changes in normalized Shannon diversity in individuals ≤ 50 years and > 50 years. **(h)** Age-associated decline in normalized Shannon diversity shown separately for individuals ≤ 50 years and > 50 years, stratified by sex and CMV status, with linear fits and 95% CIs. **(i)** Forest plot showing effect estimates for age-group-, sex-, and CMV-associated changes in normalized Shannon diversity in individuals ≤ 50 years and > 50 years.

**Figure S4:** Age-associated shifts in TCR clonal expansion across cohorts. (**a)** Stacked bar plots showing the relative distribution of T cell clone size categories across age groups within each cohort comprising individuals without cancer. **(b)** Boxplots showing the percentage of hyper-expanded clones across age groups, stratified by CMV status, based on the cohorts comprising individuals without cancer generated using the ImmunoSEQ assay. p-values were calculated using the Wilcoxon rank-sum test (****p < 0.0001). **(c)** Absolute difference in the proportion of hyper-expanded clones between CMV+ and CMV individuals across age groups, based on the cohorts comprising individuals without cancer generated using the ImmunoSEQ assay.

**Figure S5:** Age-, sex-, CMV- and cancer-associated changes in TCR repertoire diversity and clonal expansion. **(a)** Normalized Shannon diversity as a function of age within each cancer cohort. GAM fits with 95% confidence intervals are shown, stratified by sex (female, pink; male, blue; unknown, gray). Spearman correlation coefficients and corresponding p-values are indicated, and sample sizes are provided for each cohort. **(b)** Forest plot showing regression coefficients from a linear model fitted to individuals over 50 years of age. The plot summarizes associations between age group, sex, CMV status, cancer status, and normalized Shannon diversity. **(c)** Forest plot showing regression coefficients from a linear model fitted to individuals over 50 years of age, including an interaction between CMV and cancer status and adjustment for age group and sex. Analyses in (b) and (c) were restricted to cohorts generated using the ImmunoSEQ assay. Regression coefficients represent the estimated difference in normalized Shannon diversity for the category indicated in brackets relative to its reference category. The age-group coefficient represents the estimated change in normalized Shannon diversity per 10 years, while the interaction coefficient represents the additional association between CMV positivity and cancer status beyond their individual associations. **(d)** Stacked bar plots showing the relative distribution of T cell clone size categories across age groups within each cancer cohort. Sample sizes are indicated below each bar.

**Figure S6:** Associations of age, sex, CMV status, cancer status, and the interaction between CMV and cancer status with clonal size distribution. Forest plots showing regression coefficients from linear models for the percentage of (a) hyper-expanded, (b) intermediate-expanded, (c) low-expanded, and (d) non-expanded TCR clones. Models included age group, sex, CMV status, cancer status, and an interaction between CMV and cancer status. Regression coefficients represent estimated changes in percentage points. For categorical predictors, estimates are relative to the corresponding reference categories, while the interaction coefficient represents the additional association between CMV positivity and cancer status.

**Figure S7:** Prognostic impact of CMV status on overall survival. Forest plot summarizing Cox proportional hazards regression analyses assessing the association between CMV status and OS across independent cancer cohorts. Hazard ratios are shown for CMV+ individuals relative to CMV– individuals.

**Figure S8:** Prognostic impact of TCR diversity and T cell fraction across TCGA cancer types. (a–b) Forest plots summarizing Cox proportional hazards regression analyses adjusted for age and sex, assessing the association between OS and (a) normalized TCR repertoire diversity groups and (b) T cell fraction groups across TCGA cancer types. Groups were defined using the 25th percentile threshold (bottom 25% vs. top 75%), with the top 75% serving as the reference group.

**Figure S9:** CMV status classification model**. (a)** Scatter plot showing the relationship between the number of unique TCR-β clones and CMV-associated TCR-β clones, stratified by CMV status in the Emerson cohort. **(b)** Receiver operating characteristic (ROC) curves illustrating classifier performance in distinguishing CMV+ and CMV– individuals in the training and validation cohorts.

| Cohort | Sample size | Cohort characteristics | Material | Method | Sample timing |
| --- | --- | --- | --- | --- | --- |
| Gittelman | 2491 | Healthy (Population cohort, some COVID-19) | DNA | ImmunoSEQ |  |
| Nolan | 1055 | Healthy (COVID-19) | DNA | ImmunoSEQ |  |
| Emerson | 674 | Healthy | DNA | ImmunoSEQ |  |
| Delmonte | 320 | Healthy (COVID-19) | DNA | ImmunoSEQ |  |
| Mitchell | 357 | Healthy (pre-/at-onset Type 1 diabetes + at-risk healthy controls) | DNA | ImmunoSEQ |  |
| Hamm | 145 | 88 healthy, 19 lung cancer, 19 colorectal cancer, 19 head and neck cancer | DNA | ImmunoSEQ | Pretreatment (patients with cancer) |
| Pothuri | 157 | Pancreatic ductal adenocarcinoma (PDAC) | DNA | ImmunoSEQ | Collected during surgical resection, patients received chemotherapy, chemoradiotherapy, or no neoadjuvant therapy |
| Reuben | 120 | Non-small cell lung cancer (NSCLC) | DNA | ImmunoSEQ | Pretreatment |
| Snyder | 29 | Urothelial carcinoma | DNA | ImmunoSEQ | Pretreatment ICI; previously treated |
| Valpione | 28 | Melanoma | DNA | ImmunoSEQ | Pretreatment |
| Kjær | 119 | Muscle-invasive bladder cancer (MIBC) | DNA | Illumina TCR Beta-SR panel | Pretreatment |
| Nishida | 233 | Breast cancer | RNA | RNase H-dependent PCR-enabled T Cell Receptor sequencing (rhTCRseq) protocol | Pretreatment |
| TRACERx | 55 | NSCLC | RNA | Illumina Nextera XT kit | Pretreatment |
| Britanova | 67 | Healthy | RNA | 5′ template-switching protocol |  |

**Table S1:** Overview of cohorts included in this study**.** Summary of all cohorts used for TCR repertoire analyses, including sample size, disease context, input material, sequencing method and sample timing.

| Age group | Sex | Sample size | P-value |
| --- | --- | --- | --- |
| 0-10 | Female | 140 | 1.41e-07 |
| 0-10 | Male | 173 | 2.51e-08 |
| 11-20 | Female | 169 | 1.30e-08 |
| 11-20 | Male | 213 | 1.81e-11 |
| 21-30 | Female | 280 | 2.01e-06 |
| 21-30 | Male | 248 | 6.55e-04 |
| 31-40 | Female | 305 | 2.31e-10 |
| 31-40 | Male | 287 | 1.25e-04 |
| 41-50 | Female | 368 | 5.33e-09 |
| 41-50 | Male | 367 | 4.79e-09 |
| 51-60 | Female | 412 | 3.82e-11 |
| 51-60 | Male | 451 | 1.28e-08 |
| 61-70 | Female | 299 | 1.94e-07 |
| 61-70 | Male | 379 | 7.76e-10 |
| 71-80 | Female | 233 | 7.16e-06 |
| 71-80 | Male | 256 | 9.96e-12 |
| 81-90 | Female | 178 | 5.56e-07 |
| 81-90 | Male | 145 | 7.05e-02 |

**Table S2:** Comparison of TCR repertoire diversity by CMV status across age groups and sexes. Wilcoxon rank-sum test p-values comparing normalized Shannon diversity between individuals predicted to be CMV+ and CMV– within each age group, stratified by sex.

| Age group | Clone size | P-value |
| --- | --- | --- |
| 41-50 | Non-expanded | 0.791 |
| 41-50 | Hyper-expanded | 0.179 |
| 51-60 | Non-expanded | 0.0226 |
| 51-60 | Hyper-expanded | 0.527 |
| 61-70 | Non-expanded | 0.0469 |
| 61-70 | Hyper-expanded | 0.550 |
| 71-80 | Non-expanded | 0.000450 |
| 71-80 | Hyper-expanded | 0.00424 |
| 81-90 | Non-expanded | 0.355 |
| 81-90 | Hyper-expanded | 0.511 |

**Table S3:** Cancer-associated differences in TCR clone size distributions across age groups. Wilcoxon rank-sum test p-values comparing the proportions of non-expanded and hyper-expanded TCR clones between patients with cancer and individuals without cancer within each age group.

| Cohort | Samples excluded |
| --- | --- |
| Britanova | 28 |
| Hamm | 3 |
| TRACERx | 3 |
| Reuben | 0 |
| Nishida | 48 |
| Pothuri | 0 |
| Snyder | 0 |
| Valpione | 1 |
| Gittelman | 0 |
| Nolan | 1 |
| Delmonte | 1 |
| Mitchell | 2 |
| Emerson | 1 |

**Table S4:** Cohort-specific sample exclusions following downsampling**.** Samples generated using UMI-based sequencing were excluded if they contained fewer than 5000 TCR-β clones. The table summarizes the number of excluded samples in each cohort.
